# Anxiolytic Effect of Carvedilol in Chronic Unpredictable Stress Model

**DOI:** 10.1155/2022/6906722

**Published:** 2022-08-19

**Authors:** Caren Nádia Soares de Sousa, Ingridy da Silva Medeiros, Germana Silva Vasconcelos, Gabriel Ângelo de Aquino, Francisco Maurício Sales Cysne Filho, Jamily Cunha de Almeida, Ana Paula Negreiros Nunes Alves, Danielle S. Macêdo, Luzia Kalyne Almeida Moreira Leal, Silvânia Maria Mendes Vasconcelos

**Affiliations:** ^1^Neuropsychopharmacology Laboratory, Drug Research and Development Center, Department of Physiology and Pharmacology, Faculty of Medicine, Federal University of Ceará, Fortaleza, Ceará, Brazil; ^2^Histopathology Laboratory, Drug Research and Development Center, Department of Physiology and Pharmacology, Faculty of Medicine, Federal University of Ceará, Fortaleza, Ceará, Brazil; ^3^Center for Pharmaceutical and Cosmetic Studies, Faculty of Pharmacy, Dentistry and Nursing, Department of Pharmacy, Federal University of Ceará, Fortaleza, Ceará, Brazil

## Abstract

Anxiety disorders are the most prevalent psychiatric disorders being also a comorbid state of other diseases. We aimed to evaluate the anxiolytic-like effects of carvedilol (CVD), a drug used to treat high blood pressure and heart failure with potent antioxidant effects, in animals exposed to chronic unpredictable stress (CUS). To do this, female Swiss mice were exposed to different stressors for 21 days. Between days 15 and 21, the animals received oral CVD (5 or 10 mg/kg) or the antidepressant desvenlafaxine (DVS 10 mg/kg). On the 22^nd^ day, behavioral tests were conducted to evaluate locomotor activity (open field) and anxiety-like alterations (elevated plus-maze—EPM and hole board—HB tests). After behavioral determinations, the animals were euthanized, and the adrenal gland, blood and brain areas, prefrontal cortex (PFC), and hippocampus were removed for biochemical analysis. CUS reduced the crossings while increased rearing and grooming, an effect reversed by both doses of CVD and DVS. CUS decreased the number of entries and permanence time in the open arms of the EPM, while all treatments reversed this effect. CUS reduced the number of head dips in the HB, an effect reversed by CVD. The CUS reduced weight gain, while only CVD5 reversed this effect. A reduction in the cortical layer size of the adrenal gland was observed in stressed animals, which CVD reversed. Increased myeloperoxidase activity (MPO) and interferon-*γ* (IFN-*γ*), as well as reduction of interleukin-4 (IL-4) induced by CUS, were reversed by CVD. DVS and CVD increased IL-6 in both brain areas. In the hippocampus, DVS caused an increase in IFN-*γ*. Our data show that CVD presents an anxiolytic effect partially associated with immune-inflammatory mechanism regulation.

## 1. Introduction

Affective disorders, including anxiety, are associated with several comorbidities and impact the quality of life and social relationships [1–4]. In addition, these disorders are one of the leading causes of disease-related disability, especially among women.

Although anxiety etiology is not entirely understood, many theories have been proposed to explain its pathogenesis, including an interaction between biological, social, and psychological factors [5–8]. Among the risk factors for anxiety, environmental stressors are the most relevant. Indeed, environmental stressors may disrupt the hypothalamic-pituitary-adrenal axis (HPA) and contribute to the degree and duration of neuroinflammatory-driven diseases [9–12]. The chronic unpredictable stress model simulates the chronic exposure to stress factors leading to the development of major chronic mood disorders like anxiety and depression [13]. This animal model induces higher levels of cytokines in the hippocampus, like interleukin- (IL-) 1*β*, IL-6, and IL-2, and decreases motivated behavior [14]. Additionally, evidence points to the induction of peripheral oxidative and inflammatory (increase in TNF-*α*, IL-1*β*, and IL-6 inflammatory markers and a decrease in IL-10) alterations by CUS [15].

Regarding inflammatory mechanisms in anxiety, recent studies have pointed to myeloperoxidase (MPO), an enzyme mainly produced by neutrophils, as an important marker for anxiety since this enzyme is one of the links between oxidative stress and inflammation [16]. In addition, deregulation between proinflammatory and anti-inflammatory cytokines has also been found in patients with anxiety and reported as one of the mechanisms associated with the pathophysiology of anxiety [17].

Anxiety disorders are a cluster of mental disorders that include, for example, generalized anxiety disorder, social anxiety disorder (social phobia), panic, and phobias. Due to the heterogeneity of this mental disorder, the first-line interventions vary between serotonin reuptake inhibitors, cognitive behavioral therapy, and benzodiazepines. Unfortunately, less than 60% of patients respond to these treatments. Moreover, many still have residual symptoms or remain refractory to treatment; therefore, the search for new therapeutic strategies is urgent [18]. Stress-induced neuroinflammation may be involved in treatment resistance [19, 20].

Carvedilol (CVD) is a noncardioselective *β*-blocker with no negative hemodynamic and metabolic effects associated with typical blockers [21, 22]. In addition, CVD has significant antioxidant, anti-inflammatory, and cardioprotective properties in various neuropsychiatric disorders [23–28].

Despite the evidence on CVD's anxiolytic, antioxidant, and anti-inflammatory properties, no previous studies have addressed immune-inflammatory alterations as underlying mechanisms of this drug against stress-related disorders. Therefore, in this study, desvenlafaxine (DVS) was included as an antianxiety drug since it treats anxiety disorders like generalized anxiety disorder and anxiety symptoms of depression [29].

Therefore, considering CVD's antioxidant and anti-inflammatory properties [30–32], the present study is aimed at evaluating the potential anxiolytic-like effects of CVD against the CUS model, providing a rationale for the repurposing of this antihypertensive drug for the treatment of anxiety.

## 2. Material and Methods

### 2.1. Animals

Female Swiss mice (30-32g) were provided by the Drug Research and Development Center (NPDM) animal facility from the Universidade Federal do Ceará. Female mice were used due to women's higher prevalence of anxiety in relation to men [33]. Mice were kept in a controlled temperature environment (22 ± 1°C), with a standard 12-hour light/dark cycle, and received water and food ad libitum, except in periods of stress, following the experimental protocol. The study followed the ethical principles of the Brazilian College of Animal Experimentation (COBEA) and the National Council to Control Animal Experimentation (CONCEA), which comply with the international rules of scientific research involving animals [34]. The Local Ethics Committee approved the project (protocol number 26/2017).

### 2.2. Drugs

#### 2.2.1. Carvedilol

Carvedilol (CVD, Lab. Biosintética, Aché, Brazil) was diluted in distilled water and administered orally (by gavage) for seven consecutive days at a dose of 5 (CVD5) or 10 (CVD10) mg/kg in a volume of 10 ml/kg. According to the body surface area calculation [35], the doses of 5 and 10 mg/kg in mice are equivalent to the CVD's human antihypertensive doses of 12.5 and 25 mg/day, respectively.

#### 2.2.2. Desvenlafaxine

Desvenlafaxine monohydrate succinate (DVS-Lab. Wyeth, Brazil) was macerated and dissolved in distilled water and administered orally (by gavage) for seven consecutive days at 10 mg/kg in a volume of 10 ml/kg. DVS dose was determined based on previous studies from our laboratory [36, 37].

### 2.3. Experimental Protocol

Mice were subjected to varying stressors for 21 days [38], as represented in Table 1. In addition, the CUS model was used to study anxiety-like changes induced by exposure to stress [39].

**Figure 1:**
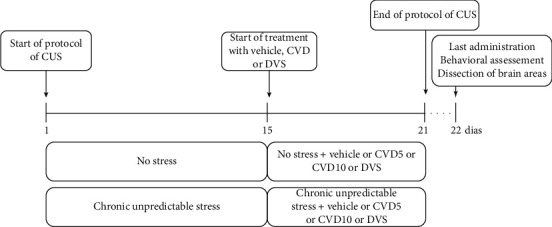
Schematic overview of the experimental design. CUS: chronic unpredictable stress; CVD: carvedilol; DVS: desvenlafaxine.

On the twenty-second day of the protocol, the animals received the last dose of water, CVD, or DVS. After 1 hour, open field, elevated plus maze, and hole board tests were conducted. In addition, an observer blinded to the experimental groups recorded the mice's behavior. Immediately after the behavioral tests, the animals were euthanized by rapid decapitation, and their adrenals, blood, and brains were removed for further neurochemical and histological analysis.  Figure 1 depicts the experimental design of the study.

To minimize the stress caused by behavioral tests and the number of animals used, the 160 animals used here were randomly distributed into two cohorts: (I) 80 animals were used to assess weight gain, open field and histological analysis of the adrenal gland, and measurement of MPO activity in the prefrontal cortex (PFC) and hippocampus; and (II) 80 animals were used to assess elevated plus maze and hole board performance, as well as corticosterone and cytokine concentrations.

Considering that the animals were randomly divided into two experimental blocks, the number of animals used between the tests varied due to the expected exclusions caused by animals that presented discrepant or stereotyped behavior throughout the experimental protocol. In addition, biochemical and histological tests also require a smaller number of animals for an accurate analysis of the data given the methodology used.

The number of animals was calculated using the “resource equation” method. This method is used when it is not possible to assume effect sizes. According to this method, the value “E” is measured based on the degree of freedom of ANOVA [40]. Based on this calculation, it was decided to use a minimum of 6 animals/group.

### 2.4. Behavioral Assessment

#### 2.4.1. Open Field Test

The open field area was made of transparent acrylic walls and a black floor (30 cm × 30 cm × 15 cm) divided into nine equal-area squares. Rearing and grooming behaviors were evaluated for five minutes [41].

#### 2.4.2. Elevated Plus-Maze Test

The elevated plus-maze test for mice consisted of two perpendicular open arms (30 × 5 cm) and two perpendicular closed arms (30 × 5 × 25 cm). The open and closed arms were connected by a central platform (5 × 5 cm). The maze was 45 cm above the floor. Each animal was placed at the center of the apparatus and was observed for 5 min to evaluate the following parameters: the number of entries in the open arm (NEOA), number of entries in the closed arms (NECA), the permanence time in open arms (PTOA), and permanence time in closed arms (PTCA) [42].

#### 2.4.3. Hole Board Test

This test was conducted in an apparatus with 16 evenly spaced holes with built-in infrared sensors (Ugo Basile, Brazil). In brief, the number of head dips into the holes was counted for 5 min for each animal [43].

### 2.5. Weight Measurement

Each animal's weight (g) was recorded throughout the experimental protocol every two days. Weight measurement was performed between 9 and 10 a.m. At the end of the protocol (21^st^ day), the animals' weight gain (g) was verified in relation to the first day of the experimental protocol.

### 2.6. Adrenal Gland Area and Corticosterone Evaluation

The adrenals were removed and fixed in 10% buffered formaldehyde until paraffinization. Then, the adrenals were cut and stained with hematoxylin-eosin to measure the cortical zone area of the adrenal gland. The analysis was performed using the ImageJ2 version (Wayne Rasband, National Institutes of Health, USA, disponível no endereço eletrônico http://rsbweb.nih.gov/ij/index.html.) and expressed in mm^2^.

Serum corticosterone levels were measured using a corticosterone ELISA kit (Thermo Fisher Scientific, Waltham, MA, USA). The range of the detectable level was 78,125-10,000 pg/mL, determined by absorbance of 450 nm.

### 2.7. Neurochemical Assessment

The neurochemical determinations were conducted in the prefrontal cortex and hippocampus. For the MPO assay, brain regions were immediately homogenized and tested. Then, the brain areas were stored at -80°C until assayed to measure interleukin concentrations.

#### 2.7.1. Myeloperoxidase Activity (MPO)

In a 0.5% hexadecyltrimethylammonium bromide solution, the brain areas were homogenized in 50 mM phosphate buffer (HTAB, pH 6). They were then centrifuged (40,000 g for 15 minutes at 4°C), and the MPO activity in the supernatant (0.1 mL) was measured using a spectrophotometer at 0 and 3 minutes (460 nm) [44]. For MPO units (U) calculation, we subtracted the values of the absorbances at baseline and after 3 min and calculated using a standard MPO curve. Thus, the results were expressed as U of MPO/mg of tissue.

#### 2.7.2. Interleukin- (IL-) 4, IL-6, and Interferon- (IFN-) *γ* Concentrations

The PFC and HC were homogenized in 8 volumes of PBS buffer with protease inhibitors (EMD Biosciences) and phosphatase (Sigma-Aldrich) to be later centrifuged (10,000 rpm, 5 min). The supernatant was used without dilution. According to the manufacturer's protocol, the concentration of cytokines in 50 *μ*L of samples was determined by ELISA (R&D Systems, Minneapolis, MN, USA) and expressed in pg/g of tissue.

### 2.8. Statistical Analysis

The statistical analysis was performed with GraphPad Prism 8.0 for Windows, GraphPad Software (San Diego, CA, USA). Two-way ANOVA considering the factors “treatment” (vehicle, CVD5, CVD10, and DVS) and “CUS model” (control and CUS) was used to evaluate behavioral data, MPO activity, body weight, and area of cortical and medullary adrenal zones. One-way ANOVA was used to evaluate data on cytokines and corticosterone. In both conditions, a Turkey post hoc test was performed.

Considering that no significant changes in the behavioral evaluations were observed between nonstressed mice treated with the drugs and the control group, we decided to perform the other assessments, i.e., weight changes, MPO activity, serum corticosterone, and cytokines only in the groups exposed to CUS and control, to reduce the number of animals used in the experiments.

All results are expressed as means ± S.E.M (standard error of the mean). Before ANOVA, Kolmogorov-Smirnov (distance) test was conducted to verify the normal distribution of the data. For all analyses, was considered the significance level at *α* = 0.05.

## 3. Results

### 3.1. CVD and DVS Revert Locomotor Alterations Induced by the CUS-Model

**Figure 2:**
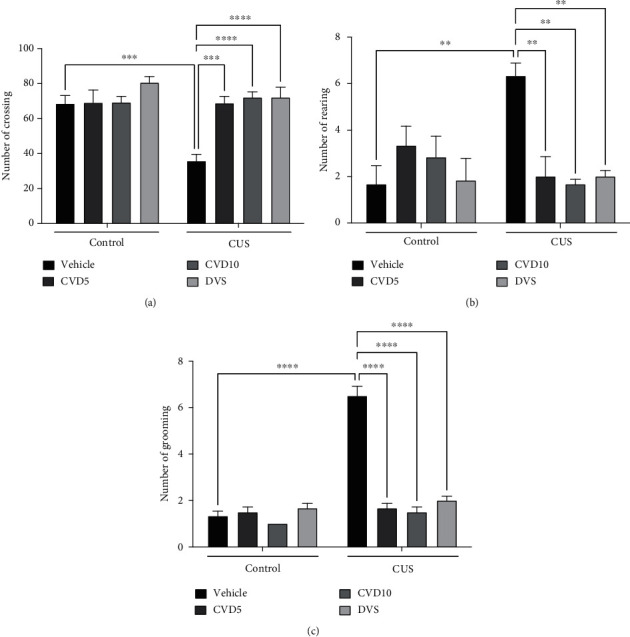
Effects of CVD and DVS treatment in the open field parameters, crossings (a), rearing (b), and grooming (c) in mice subjected to the CUS model. The animals were subjected to different stressors for 21 days. Between the 15^th^ and 21^st^ days of the stress protocol, mice received CVD (5 or 10 mg/kg, p.o.) or DVS (10 mg/kg, p.o.). On the 22^nd^ day, the animals were submitted to the open field test, 1 h after administering drugs, without applying stressors. Each column represents the mean ± SEM (*n* = 6 − 9 animals/group). ^∗∗^*P* < 0.01, ^∗∗∗^*P* < 0.001, and ^∗∗∗∗^*P* < 0.0001 according to Tukey's multiple comparison test. Abbreviations: CUS: chronic unpredictable stress; CVD: carvedilol; DVS: desvenlafaxine.

In the analysis of locomotor activity (Figure 2(a)), CUS caused a significant reduction in the number of crossings when compared with the control group (*P* < 0.001), while CVD (*P* < 0.001), in both doses, and DVS (*P* < 0.0001) reversed this effect (two-way ANOVA results: interaction (*F* (3, 40) = 5.802, *P* = 0.0022); main effect of “treatment” (*F* (3, 40) = 9.800, *P* < 0.0001); and “CUS” (*F* (1, 40) = 8.408, *P* = 0.0060)).

Besides that, rearing behavior (Figure 2(b)) increased in animals exposed to CUS in relation to control (*P* < 0.01), while all treatments reversed this alteration (*P* < 0.01) (two-way ANOVA results: interaction (*F* (3, 40) = 7.428, *P* = 0.0005); main effect of “treatment” (*F* (3, 40) = 3.158, *P* = 0.0350)).

In the evaluation of grooming behavior (Figure 2(c)), there was an increase by CUS in relation to control mice (*P* < 0.0001), while CVD at both doses and DVS significantly reversed this alteration (*P* < 0.0001) (two-way ANOVA results: interaction (*F* (3, 42) = 51.49, *P* < 0.0001); main effect of “treatment” (*F* (3, 42) = 50.58, *P* < 0.0001); and “CUS” (*F* (1, 42) = 85.28, *P* < 0.0001)).

### 3.2. CVD and DVS Reverse Anxiety-Like Behavior Induced by the CUS-Model

**Figure 3:**
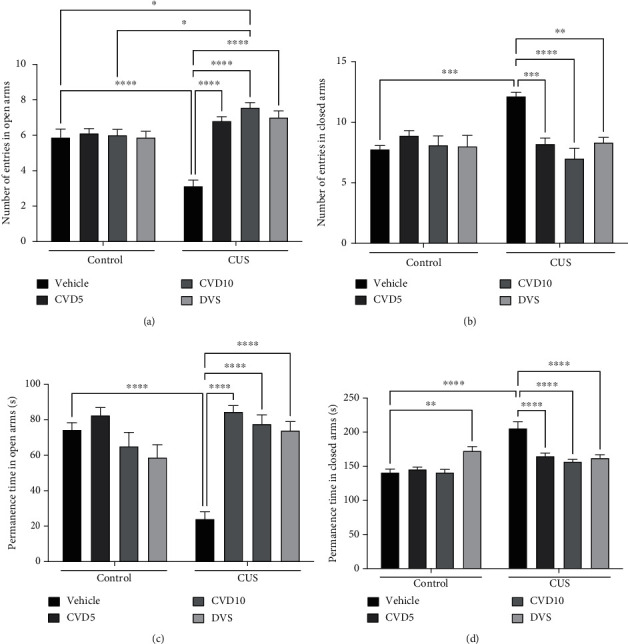
Effects of CVD and DVS treatment on plus-maze test parameters, number of entries in open arms-NEOA (a) and closed arms-NECA (b), and permanence time in open arms-PTOA (c) and closed arms-PTCA (d), in the CUS model. The animals were subjected to different stressors for 21 days. Between the 15^th^ and 21^st^ days of the stress protocol, the mice received CVD (5 or 10 mg/kg, p.o.) or DVS (10 mg/kg, p.o.). On the 22^nd^ day, the animals were submitted to the elevated plus-maze test, 1 h after administering drugs, without the application of stressors. Each column represents the mean ± SEM (*n* = 8 − 10 animals/group). ^∗^*P* < 0.05, ^∗∗^*P* < 0.01, ^∗∗∗^*P* < 0.001, and ^∗∗∗∗^*P* < 0.0001 according to Tukey's multiple comparison test. Abbreviations: CUS: chronic unpredictable stress; CVD: carvedilol; DVS: desvenlafaxine; NEOA: number of entries in open arms; NECA: number of entries in closed arms; PTOA: permanence time in open arms; PTCA: permanence time in closed arms.

CUS caused a decrease in the number of entries (Figure 3(a); *P* < 0.001) and permanence time (Figure 3(c); *P* < 0.0001) in the open arms and an increase in the number of entries (Figure 3(b); *P* < 0.0001) and permanence time (Figure 3(d); *P* < 0.0001) in the closed arms when compared with the control group. Treatment with CVD5 (NEOA: *P* < 0.0001; NECA: *P* < 0.001; PTOA: *P* < 0.0001; PTCA: *P* < 0.0001), CVD10 (NEOA: *P* < 0.0001; NECA: *P* < 0.0001; PTOA: *P* < 0.0001; PTCA: *P* < 0.0001), or DVS (NEOA: *P* < 0.0001; NECA: *P* < 0.01; PTOA: *P* < 0.0001; PTCA: *P* < 0.0001) reversed all alterations caused by CUS in this test. Interestingly, treatment with DVS in the absence of CUS increased the permanence time in the closed arms compared with control (*P* < 0.01) (two-way ANOVA results: NEOA interaction (*F* (3, 63) = 15.99, *P* < 0.0001); NECA interaction (*F* (3, 63) = 7.895, *P* = 0.0001); PTOA interaction (*F* (3, 63) = 14.44, *P* < 0.0001); and PTCA interaction (*F* (3, 63) = 15.63, *P* < 0.0001)).

**Figure 4:**
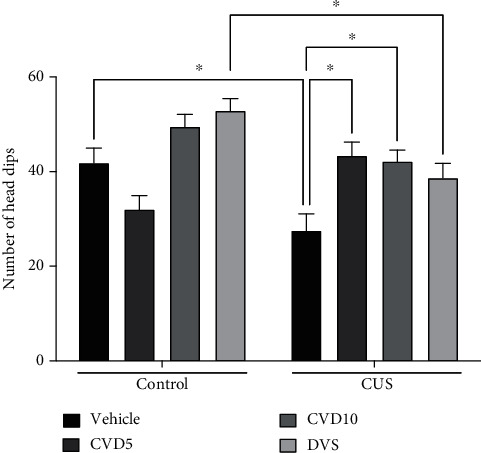
Effects of CVD and DVS treatment on hole board test in a CUS model. The animals were subjected to different stressors for 21 days. Between the 15^th^ and 21^st^ days of the stress protocol, the mice received CVD (5 or 10 mg/kg, p.o.) or DVS (10 mg/kg, p.o.). On the 22^nd^ day, the animals were submitted to the hole board test, 1 h after administering drugs, without the application of stressors. Each column represents the mean ± SEM (*n* = 6 − 8 animals/group). ^∗^*P* < 0.05 according to Tukey's multiple comparison test. Abbreviations: CUS: chronic unpredictable stress; CVD: carvedilol; DVS: desvenlafaxine.

In the analysis of the number of head dips (Figure 4), CUS exposure decreased the number of head dips in relation to the control group (*P* < 0.05). Only CVD, in both doses, reversed the alterations caused by CUS (*P* < 0.05) (two-way ANOVA results: interaction (*F* (3, 43) = 8.712, *P* = 0.0001)).

### 3.3. Effects of CUS and Anxiolytic Strategies on Body Weight

**Figure 5:**
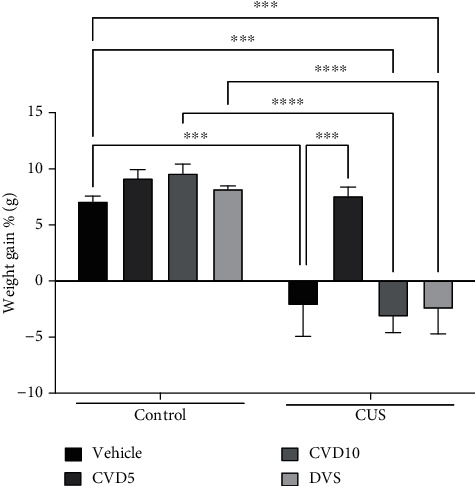
Effects of CVD and DVS treatment on weight gain in the CUS model. The animals were subjected to different stressors for 21 days. Between the 15^th^ and 21^st^ days of the stress protocol, the mice received CVD (5 or 10 mg/kg, p.o.) or DVS (10 mg/kg, p.o.). The animals' weight was recorded in grams every 2 days throughout the experimental protocol. The weight gain was calculated based on changes in body weight from day 21 relative to the bodyweight on day 1. Each column represents the weight gain ± SEM (*n* = 8 animals/group). ^∗∗∗^*P* < 0.001, and ^∗∗∗∗^*P* < 0.0001 according to Tukey's multiple comparison test. Abbreviations: CUS: chronic unpredictable stress; CVD: carvedilol; DVS: desvenlafaxine.

The CUS model reduced the mice's weight (Figure 5) gain when compared with the control (*P* < 0.001), while only the treatment with CVD5 (*P* < 0.001) reversed this effect (two-way ANOVA results: interaction (*F* (3, 56) = 5.615, *P* = 0.0019)).

### 3.4. Effects of CUS and Anxiolytic Strategies on Cortical Adrenal Zone Area and Corticosterone Levels

**Figure 6:**
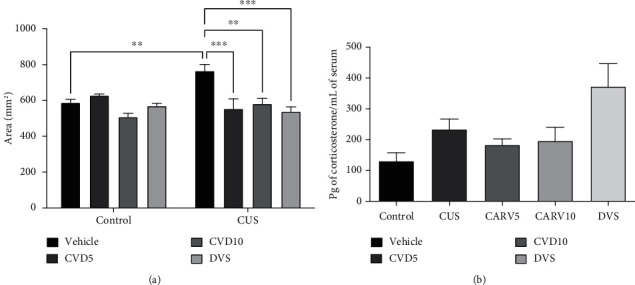
Effects of CVD and DVS treatment on adrenal cortex area (a) and corticosterone serum concentrations (b) in a CUS model. The animals were subjected to different stressors for 21 days. Between the 15^th^ and 21^st^ days of the stress protocol, the mice received CVD (5 or 10 mg/kg, p.o.) or DVS (10 mg/kg, p.o.). On the 22^nd^ day, the animals were euthanized, and their blood and adrenals were collected. Each column represents the mean ± SEM (*n* = 4 − 5 animals/group). ^∗∗^*P* < 0.01, ^∗∗∗^*P* < 0.001 according to Tukey's multiple comparison test. Abbreviations: CUS: chronic unpredictable stress; CVD: carvedilol; DVS: desvenlafaxine.

The assessment of the cortical adrenal zone area (Figure 6(a)) showed that CUS caused an increase in the cortical adrenal zone area when compared with the control group (*P* < 0.01), while CVD5 (*P* < 0.001), CVD10 (*P* < 0.01), and DVS (*P* < 0.001) reversed this effect. On the other hand, no statistically significant effect was observed in the measurement of serum corticosterone (Figure 6(b)).

### 3.5. Effects of CVD and DVS Treatment on MPO Activity

**Figure 7:**
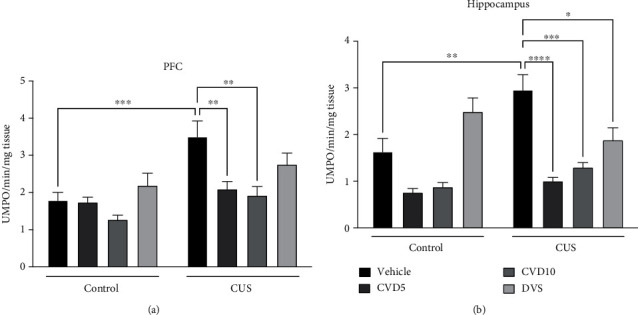
Effects of CVD and DVS treatment on myeloperoxidase (MPO) activity in the prefrontal cortex (a) and hippocampus (b). The animals were subjected to different stressors for 21 days. Between the 15^th^ and 21^st^ days of the stress protocol, the mice received CVD (5 or 10 mg/kg, p.o.) or DVS (10 mg/kg, p.o.). On the 22^nd^ day, the animals were euthanized. Each column represents the mean ± SEM (*n* = 6 − 8 animals/group). ^∗^*P* < 0.05, ^∗∗^*P* < 0.01, ^∗∗∗^*P* < 0.001, and ^∗∗∗∗^*P* < 0.0001 according to Tukey's multiple comparison test. Abbreviations: CUS: chronic unpredictable stress; CVD: carvedilol; DVS: desvenlafaxine; MPO: myeloperoxidase; PFC: prefrontal cortex; HC: hippocampus.

In the evaluation of MPO activity in the PFC (Figure 7(a)), CUS increased MPO activity in this brain area when compared with the control (*P* < 0.001), while only CVD treatment, in both doses, reversed the CUS effect (*P* < 0.01) (two-way ANOVA results: main effect of “treatment” (*F* (3, 45) = 7.357; *P* = 0.0004) and “CUS” (*F* (1, 45) = 21.03; *P* < 0.0001)).

In the analysis of MPO activity in the hippocampus (Figure 7(b)), CUS increased MPO activity in this brain area when compared with the control (*P* < 0.01), while treatment with CVD5 (*P* < 0.0001), CVD10 (*P* < 0.001), and DVS (*P* < 0.05) reversed CUS effect (two-way ANOVA results: interaction (*F* (3, 43) = 6.457; *P* = 0.0010)).

### 3.6. Effects of CVD and DVS Treatment on Brain Cytokine Concentrations

**Figure 8:**
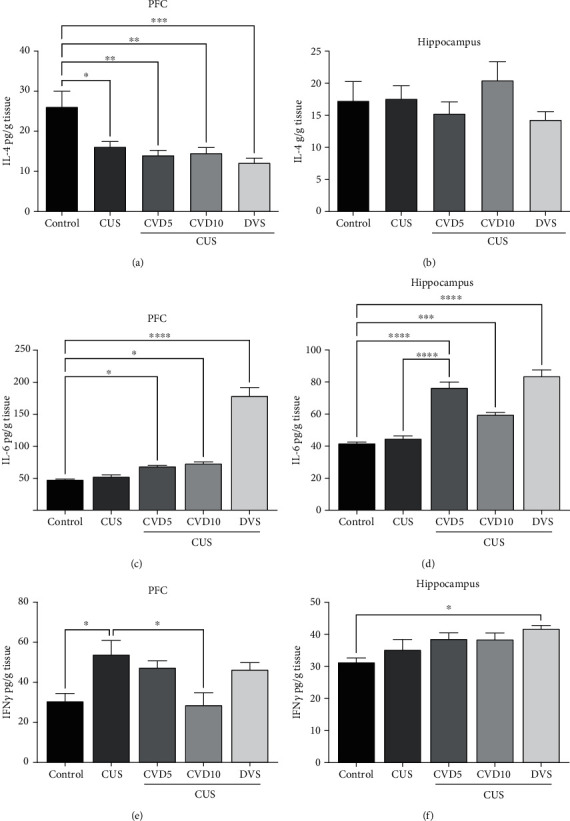
Effects of CVD and DVS treatment on IL-4 ((a) prefrontal cortex; (b) hippocampus), IL-6 ((c) prefrontal cortex; (d) hippocampus), and IFN-*γ* ((e) prefrontal cortex; (f) hippocampus) concentrations. The animals were subjected to different stressors for 21 days. Between the 15^th^ and 21^st^ days of the stress protocol, the mice received CVD (5 or 10 mg/kg, p.o.) or DVS (10 mg/kg, p.o.). On the 22^nd^ day, the animals were euthanized and had the prefrontal cortex and hippocampus dissected. Each column represents the significance ± SEM (*n* = 6 − 8 animals/group). ^∗^*P* < 0.05, ^∗∗^*P* < 0.01, ^∗∗∗^*P* < 0.001, and ^∗∗∗∗^*P* < 0.0001 according to Tukey's multiple comparison test. Abbreviations: CUS: chronic unpredictable stress; CVD: carvedilol; DVS: desvenlafaxine; IL-4: interleukin-4; IL-6: interleukin-6; IFN-*γ*: interferon-*γ*; PFC: prefrontal cortex; HC: hippocampus.

In the PFC (Figure 8(a)), CUS caused a significant decrease in IL-4 concentrations when compared with the control group (*P* < 0.01). In this area, none of the treatments reversed the effect caused by CUS.

On the other hand, no changes were observed in the hippocampus (Figure 8(b)).

CUS did not alter IL-6 levels in the PFC (Figure 8(c)) in relation to the control group. However, treatment with DVS (*P* < 0.0001), CVD5 (*P* < 0.05), or CVD10 (*P* < 0.05) significantly increased IL-6 concentrations when compared to control group.

Like the effects in the PFC, CUS did not cause any change in IL-6 concentrations in the hippocampus (Figure 8(d)) compared with the control group. However, all treatments significantly increased the concentrations of this cytokine when compared with the control and CUS groups (*P* < 0.001).

In the PFC (Figure 8(e)), CUS caused a significant increase in IFN-*γ* concentrations when compared with the control group (*P* < 0.01). Only the CVD10 treatment reversed the effect caused by CUS (*P* < 0.05). CUS did not cause changes in the concentrations of IFN-*γ* in the hippocampus (Figure 8(f)) when compared with the control group (*F* (4, 25) = 3.699; *P* = 0.0169). On the other hand, treatment with DVS induced higher concentrations of this cytokine compared with the control group (*P* < 0.05).

## 4. Discussion

In the present study, CUS induced behavioral alterations that mimic anxiety in apparatus that are well-validated for this end, namely, plus-maze and role board tests [36, 45–48]. In this regard, we observed decreased number of entries and time spent in the open arms, increased entries and permanence time in the closed arms of the plus-maze, and decreased head dips in the role board test. Additionally, we observed reduced weight gain, increased adrenal area, and proinflammatory alterations in brain areas related to mood regulation, namely, PFC and hippocampus. The administration of CVD reversed all behavioral and some neuroinflammatory alterations induced by CUS and weight gain changes. On the other hand, DVS, used here as an antidepressant drug to treat anxiety disorders, could not reverse weight gain and some proinflammatory alterations induced by CUS. Our results revealed that CVD might be an important strategy for treating anxiety disorders.

Repeated exposure to stress induces adaptive changes in the brain involving the cumulative action of glucocorticoids that facilitate the development of stress-related psychiatric disorders, including anxiety [49]. For example, anxiety disorders are marked by excessive fear and autonomic nervous system activity changes, like increased heart rate and blood pressure [50]. To date, antidepressant drug treatment is the clinical standard for all anxiety disorders [51].

In the present study, we performed the open field test besides performing validated tests to evaluate anxiety-like behaviors in rodents, namely, plus-maze and role board tests, to expand the behavioral assessment. We observed that CUS caused a reduction in locomotor activity (evaluated by the number of crossings), a behavioral alteration already reported in different animal models of anxiety [36, 52, 53]. Psychomotor retardation is an important symptom of generalized anxiety disorder [54–56]. Other important parameters measured in the open field test and relevant for anxiety were rearing and grooming behaviors. Indeed, previous findings have shown a relationship between altered rearing and anxiety in animal models [57, 58]. Regarding grooming, we observed higher levels in CUS-exposed animals. This parameter is extensively investigated in animal models of anxiety [29, 59, 60]. Our results, therefore, reinforce the idea that CUS causes anxiety-like behavior.

We observed that both CVD and DVS improved psychomotor performance and anxiety-related parameters evaluated in the open field test [56, 61]. It deserves to be mentioned that the anxiolytic effect of DVS is already well established in the literature [29, 36, 62], which is not true for CVD.

Accumulated evidence reveals that stress is associated with the activation of neuroendocrine pathways culminating in the development of anxiety disorders. One of the mechanisms related to this dysregulation is the activation of beta-adrenergic receptors in glial cells and some brain regions. Thus, the blockade of these receptors seems to be an important anxiolytic strategy already observed in animal models and human studies [63–67]. Indeed, we observed that CVD was superior to DVS in regulating anxiety-like alterations induced by CUS, probably due to its nonselective blockade of adrenergic receptors.

Weight changes are often observed in patients with depression and anxiety, and this symptom is considered for the diagnostic classification [54]. In this regard, the literature has already documented that stress promotes the mobilization of the body's energy reserves as an adaptation mechanism to respond to threatening situations. Furthermore, stress-induced hyperactivation of the HPA axis and the sympathetic nervous system causes a positive and dialectical regulatory mechanism between these two systems associated with anorexia hypophagia and weight loss [68, 69].

Our findings showed that CVD5 reversed the weight changes caused by CUS. This effect is probably associated with the adrenergic blocking action of CVD, considering that the stimulation of lipolysis and the release of fatty acids from adipose tissue and glycogenolysis and gluconeogenesis in the liver are activities regulated by alpha- and beta-adrenergic receptors [70, 71]. However, this appears to be a dose-dependent effect, as only the lowest dose reversed the weight loss caused by CUS.

In line with stress deregulation of the HPA axis [72, 73], CUS increased the adrenal cortical layer area. However, this alteration was reversed by both CVD and DVS. This effect is probably related to a regulation of stress-induced adrenocorticotropic hormone (ACTH) stimulation on cells in this region, especially the glomerulus layer [72, 74].

Given the importance of proinflammatory alterations as underlying mechanisms of anxiety, we decided to investigate the activity of MPO and the levels of pro- and anti-inflammatory cytokines in the PFC and hippocampus. We observed that CUS induced increased activity of MPO in the PFC and hippocampus, which was reversed in both brain areas by the two doses of CVD and only in the hippocampus by DVS. MPO is produced by leukocytes [75–77], representing an important link between these oxidative mechanisms and inflammation [78]. Furthermore, this enzyme is an important biomarker for mental disorders [76].

Previous studies indicate that the microglial reactivity observed in stressful situations is associated with noradrenaline's direct activation of beta-adrenergic receptors. This beta-adrenergic activation of microglia cells contributes to synthesizing and releasing inflammatory mediators and oxidative stress [75, 79, 80]. Since CVD is a nonselective beta-blocker with alpha-blocker properties, this drug seems to present advantages for treating neuropsychiatric disorders associated with a dysregulation in adrenergic and inflammatory mechanisms.

Among the cytokines involved in the pathophysiology of anxiety, IL-4 stands out for its anti-inflammatory activity. Here, we observed that CUS caused a reduction in IL-4 concentrations in the PFC. This result is in line with studies indicating that low concentrations of IL-4 are associated with anxiety symptoms [81]. However, in our experimental conditions, the drugs could not reverse CUS-induced alterations in IL-4. Therefore, the observed absence of effects may probably be related to the short administration time of these drugs (only 7 days).

Besides being related to the neurobiology of anxiety [49, 82, 83], in our results, we did not observe CUS-induced alterations in IL-6 levels in the PFC, nor in the hippocampus. Furthermore, several studies indicate that the chronic stress protocol of 28 days or more can be more robust when mimicking a greater variety of neurochemical and behavioral changes, including changes in brain cytokines. Finally, it is worth noting that the stressors used in the protocol and the sex of the animals can influence the observed findings [84–86] since females are less prone to developing brain inflammatory alterations compared with males [87].

On the other hand, treatments with CVD or DVS increased IL-6 concentrations in relation to control animals. This is an intriguing result that must be further explored. Indeed, the cellular expression of the membrane-bound and soluble forms of IL-6R (Interleukin-6 receptor) and its receptor, gp130 (Glycoprotein 130) is modulated by many factors, including proteases, cytokines, chemical drugs, and intracellular signaling pathways, contributing to IL-6 pro- and anti-inflammatory effects [88]. In addition, IL-6 regulates the HPA axis, and possibly the alteration observed here is related to this effect [49].

Several studies have reported high circulating levels of IFN-*γ* in anxiety patients [89, 90]. In addition, some anxiolytics can antagonize IFN-*γ* signaling [91–93].

Here, CVD10 reversed the increase in IFN-*γ* caused by CUS in the PFC. Corroborating this idea, previous findings indicate that treatment with selective serotonin reuptake inhibitors decreases the expression of IFN-*γ* mRNA in patients with anxiety [94]. Despite this, in our results, DVS did not reverse the CUS-induced increase in PFC IFN-*γ* levels but instead increased the levels of this cytokine in the hippocampus. This result is divergent from the literature and requires further investigation.

Clinical and preclinical studies have already reported the anxiolytic effect of CVD, strengthening the data presented here [63, 64, 66, 67]. However, studies addressing neuroinflammatory mechanisms underlying the action of this drug are still scarce, mainly using stress models of anxiety.

## 5. Conclusion

The present study is the first to demonstrate that anti-inflammatory mechanisms underlie CVD anxiolytic action in an animal model of anxiety/depression induced by CUS. We also showed here that the effect of CVD was superior to DVS in some parameters, revealing that the nonselective blockade of adrenergic receptors combined with antioxidant and possibly immunoregulatory effects of this drug is relevant for the reversal of behavioral and neurobiological alterations induced by repeated stress exposure. Besides that, cardiovascular changes like hypertension or heart failure are commonly observed in anxiety patients emphasizing the repurposing of CVD as a therapeutic alternative for patients with depression and anxiety, therefore reducing the need for polypharmacy and the side effects observed in the current pharmacotherapy.

## Figures and Tables

**Table 1:** Daily stressors used in chronic unpredictable stress induction. Adapted from Kumar, Kuhad, and Chopra [38].

Day	Time/period	Stressors
1^st^	5 min/morning	Swimming in cold water (12°C)
2^nd^	30s/afternoon	Tail pinch
3^rd^	24 h/night	Water and food deprivation
4^th^	12h/night	Night lighting
5^th^	—	No stress
6^th^	15 min/morning	Swimming in water at room temperature (23 ± 2°C)
7^th^	60s/afternoon	Tail pinch
8^th^	5 min/morning	Swimming in cold water (12°C)
9^th^	12h/night	Night lighting
10^th^	—	No stress
11^th^	10 min/morning	Swimming in water at room temperature (23 ± 2°C)
12^th^	90s/afternoon	Tail pinch
13^th^	5 min/morning	Swimming in cold water (12°C)
14^th^	24 h/afternoon	Water and food deprivation
15^th^	12h/night	Night lighting
16^th^	—	No stress
17^th^	24 h/morning	Water and food deprivation
18^th^	60s/afternoon	Tail pinch
19^th^	15 min/morning	Swimming in water at room temperature (23 ± 2°C)
20^th^	12 h/night	Night lighting
21^st^	5 min/morning	Swimming in cold water (12 °C)

## Data Availability

The behavioral and neurochemical data used to support the findings of this study are included within the article.
